# A248 OUTCOMES OF COVID-19 ILLNESS AND ACCESS TO APPROPRIATE TREATMENT IN LIVER TRANSPLANT RECIPIENTS IN BRITISH COLUMBIA THROUGHOUT THE PANDEMIC

**DOI:** 10.1093/jcag/gwac036.248

**Published:** 2023-03-07

**Authors:** J Reid, E Yoshida, T Hussaini, J -A Harrigan

**Affiliations:** 1 Division of Gastroenterology; 2 Department of Pharmacy; 3 Department of Nursing, University of British Columbia, Vancouver, Canada

## Abstract

**Background:**

COVID-19 continues to cause significant illness and mortality worldwide. Solid Organ Transplant Recipients (SOTR) have a higher rate of COVID-19 infection and worse outcomes than those who are immunocompetent. Dexamethasone, tocilizumab, and baricitinib have improved inpatient outcomes. Sotrovimab, remdesivir, and nirmatrelvir/ritonavir have recently been approved for used in high risk, minimally symptomatic outpatients. Previous experience has shown that use of monoclonal antibodies and oral antiviral agents have reduced morbidity and mortality of COVID-19 in SOTR.

**Purpose:**

To assess the experiences and outcomes of COVID-19 and access to directed therapy in SOTR in British Columbia (BC).

**Method:**

Data was compiled from patient disclosure to liver transplant clinicians on COVID-19 infection and gathered from patient charts in the SOTR Clinic at Vancouver General Hospital. Inclusion criteria were patients followed at the clinic with a positive COVID-19 test or clinical confirmation of COVID-19 syndrome. This is a retrospective, quality assurance study and did not require ethics review.

**Result(s):**

158 SOTR reported COVID-19 infections between March 2020 and September 2022. 3 patients died within 30 days of COVID-19 infection, 2 (1.26%) of which the cause of death was directly due to COVID-19, and the other who had cholangitis with severe sepsis and multi-organ system failure. 24 patients required admission to hospital, 7 requiring critical care support.

41 patients did not receive any therapy for COVID-19: there was none available at that time (n=26), it was contraindicated due to a drug interaction (n=2) or medical condition (n=1), was refused (n=1), or the infection was reported too late to qualify (n=10). 83% (92/112) of outpatients received available anti-viral treatment: sotrovimab (n=27), remdesivir (n=63), or nirmatrelvir/ritonavir (n=2). In inpatients (n=24), 13 received corticosteroids, 6 dual treated with tocilizumab (n=4) or baracitinib (n=2). 4 inpatients received remdesivir.

Three patients were treated in hospital after initiating outpatient therapy, one with progression of COVID-19 illness despite starting remdesivir. Two patients had adverse effects of medications provided: one was prescribed nirmatrelvir/ritonavir by a physician outside of the transplant program, which caused tacrolimus toxicity (serum concentration of 69.4 ng/mL) with nausea, vomiting, and diarrhea. Another patient had an episode of hypotension after receiving sotrovimab and sustained an acute kidney injury (AKI). Both patients fully recovered. There were no deaths on antiviral therapy.

Of 145 patients who had laboratory investigations done within 30 days of COVID-19 infection, 16 had a transient rise in liver enzymes, 14 had an AKI and 11required an adjustment in their tacrolimus dose.

**Conclusion(s):**

Involving the liver transplant team early in the course of COVID-19 illness allows patients to safely access COVID-19 directed therapy to avoid progression of illness, and medication interactions or toxicity.

**Please acknowledge all funding agencies by checking the applicable boxes below:**

None

**Disclosure of Interest:**

None Declared

